# An Efficient Score Test Integrated with Empirical Bayes for Genome-Wide Association Studies

**DOI:** 10.3389/fgene.2021.742752

**Published:** 2021-10-01

**Authors:** Jing Xiao, Yang Zhou, Shu He, Wen-Long Ren

**Affiliations:** Department of Epidemiology and Medical Statistics, School of Public Health, Nantong University, Nantong, China

**Keywords:** computational efficiency, score test, empirical bayes, linear mixed model, genome-wide association studies, multi-locus

## Abstract

Many methods used in multi-locus genome-wide association studies (GWAS) have been developed to improve statistical power. However, most existing multi-locus methods are not quicker than single-locus methods. To address this concern, we proposed a fast score test integrated with Empirical Bayes (ScoreEB) for multi-locus GWAS. Firstly, a score test was conducted for each single nucleotide polymorphism (SNP) under a linear mixed model (LMM) framework, taking into account the genetic relatedness and population structure. Then, all of the potentially associated SNPs were selected with a less stringent criterion. Finally, Empirical Bayes in a multi-locus model was performed for all of the selected SNPs to identify the true quantitative trait nucleotide (QTN). Our new method ScoreEB adopts the similar strategy of multi-locus random-SNP-effect mixed linear model (mrMLM) and fast multi-locus random-SNP-effect EMMA (FASTmrEMMA), and the only difference is that we use the score test to select all the potentially associated markers. Monte Carlo simulation studies demonstrate that ScoreEB significantly improved the computational efficiency compared with the popular methods mrMLM, FASTmrEMMA, iterative modified-sure independence screening EM-Bayesian lasso (ISIS EM-BLASSO), hybrid of restricted and penalized maximum likelihood (HRePML) and genome-wide efficient mixed model association (GEMMA). In addition, ScoreEB remained accurate in QTN effect estimation and effectively controlled false positive rate. Subsequently, ScoreEB was applied to re-analyze quantitative traits in plants and animals. The results show that ScoreEB not only can detect previously reported genes, but also can mine new genes.

## Introduction

Genome-wide association studies (GWAS) have become a powerful approach in the genetic dissection of quantitative traits in human, animal and plant genetics (Buniello et al., 2019; Jiang et al., 2019). A number of statistical methods for GWAS have been developed to facilitate the discovery of potentially associated genetic variants. Linear mixed model (LMM) approaches have been widely used due to the capacity to correct genetic relatedness and population structures, thereby minimizing false positives (Zhang et al., 2005; Yu et al., 2006; Aulchenko et al., 2007). Consequently, the number of LMM-based computational tools for genetic studies is rapidly increasing, and includes efficient mixed model association (EMMA) (Kang et al., 2008), a compressed MLM with population parameters previously determined (P3D) (Zhang et al., 2010), factored spectrally transformed linear mixed models (FaST-LMM) (Lippert et al., 2011), genome-wide complex trait analysis (GCTA) (Yang et al., 2011), genome-wide efficient mixed model association (GEMMA) (Zhou and Stephens, 2012), BOLT-LMM (Loh et al., 2015), and the rapid and efficient linear mixed model approach using the score test (LMM-Score) (Chang et al., 2019b). Although these methods have successfully detected a number of variants among various traits, they still have some shortcomings. Most adopt single-locus screening, so that the combined effects of multiple loci are ignored and the threshold in multiple test correction is often difficult to determine (Wang et al., 2016; Ren et al., 2018; Wen et al., 2018).

Several classical approaches have been proposed to address these issues, such as, the least absolute shrinkage and selector operator (Lasso) (Tibshirani, 1996), Elastic-Net (Zou and Hastie, 2005), Bayesian Lasso (Park and Casella, 2008), and Empirical Bayes (Xu, 2010). These approaches have been shown to perform better than single-locus approaches, but most are computationally unfeasible in GWAS. It brings great challenge when the number of predictors is significantly larger than the number of observations. An available solution is to perform dimensionality reduction prior to variable selection. For example, the multi-locus random-SNP-effect mixed linear model (mrMLM) uses the Wald test based on a random-SNP-effect linear mixed model to reduce dimensionality, then, all the selected markers are placed into a multi-locus model, showing advantage in controlling complex population structure (Wang et al., 2016), integration of the Kruskal-Wallis test with Empirical Bayes with polygenic background control (pKWmEB) uses the non-parametric Kruskal-Wallis test to perform initial screening of all SNPs, which is more powerful in the case in which the phenotypic value violates the assumption of a normal distribution (Ren et al., 2018), fast multi-locus random-SNP-effect EMMA (FASTmrEMMA) first chooses all putative quantitative trait nucleotides (QTNs) with *p*-values ≤ 0.005 and then includes them in a multi-locus model for true QTN detection (Wen et al., 2018), iterative modified-sure independence screening EM-Bayesian lasso (ISIS EM-BLASSO) uses an iterative modified-sure independence screening (ISIS) approach in reducing the number of SNPs to a moderate size, and next estimates all the selected SNP effects in the reduced model (Tamba et al., 2017), hybrid of restricted and penalized maximum likelihood (HRePML) performs restricted maximum likelihood on single-locus LMM to remove unrelated markers, and then carries out penalized maximum likelihood to select true QTN (Ren et al., 2020), a fast Empirical Bayes method (Fast-EB-LMM) uses a modified kinship matrix accounting for individual relatedness to avoid competition between the locus of interest and its counterpart in the polygene (Chang et al., 2019a), and multi-locus mixed-model (MLMM) adopts stepwise mixed-model regression with forward inclusion and backward elimination, and handles the confounding effects of large numbers of loci well (Segura et al., 2012). Although these multi-locus methods have achieved good results in many GWAS analyses, their computational efficiency is not very satisfactory.

Fortunately, the score test can greatly decrease computational time. Furthermore, a major advantage of the score test is that it only requires imputation under the null model of no association, and working within the framework of the score test makes other extensions feasible. Xiong et al. (2002) proposed a generalized Hotelling’s T2 test for the analysis of quantitative and qualitative traits, and Wallace et al. (2006) extended it to a marker-based score test for linkage disequilibrium mapping by selective genotyping. To further improve computational efficiency, Tang et al. (2009) developed a principal component-based score test within a variable-sized sliding-window. To incorporate additional phenotypic information of relatives who are not genotyped, Thornton and McPeek (2007) proposed the more powerful quasi-likelihood score (MQLS) test. Uh et al. (2009) extended the MQLS to the genotypic MQLS (gMQLS) test to accommodate different genetic models. However, the aforementioned score tests are unable to estimate quantitative trait nucleotide (QTN) effects, and are weak in controlling confounding.

In this study, we proposed an efficient association analysis approach by integrating the score test with Empirical Bayes, named ScoreEB, under the framework of the mrMLM (Wang et al., 2016) and FASTmrEMMA (Wen et al., 2018) approaches. Firstly, the score test is performed to select all of the markers that are potentially associated with the trait, taking into account the genetic relatedness and population structure within the linear mixed model. The mixed model equations were solved using preconditioned conjugate gradient iteration (PCG), which requires only performing matrix-vector products. The PCG algorithm is one of the best known iterative methods for solving linear systems with symmetric, positive definite matrix (Legarra and Misztal, 2008; VanRaden, 2008). Secondly, all of the selected markers are placed into a multi-locus model and their effects are estimated by Empirical Bayes. Then, all of the nonzero effects are further identified by a likelihood ratio test. Our new method ScoreEB adopts the similar strategy of mrMLM (Wang et al., 2016) and FASTmrEMMA (Wen et al., 2018), and the only difference is that we use the score test to select all the potentially associated markers. ScoreEB fills the gap between existing multi-locus and single-locus method, and it not only has high computational efficiency, but also can control confounding well. To validate the effectiveness of our method, we compare it with other five methods, mrMLM (Wang et al., 2016), FASTmrEMMA (Wen et al., 2018), ISIS EM-BLASSO (Tamba et al., 2017), HRePML (Ren et al., 2020), and GEMMA (Zhou and Stephens, 2012) using a series of simulation studies and real data analysis in plants and animals.

## Methods

### Genetic Model

#### Random QTN Effect Linear Mixed Model

A conventional linear mixed model used for association testing can be expressed as

y=Xb+xβ+u+ε (1)

where ***y*** denotes an *n* × 1 quantitative phenotype vector for *n* individuals; ***X*** denotes the *n* × *c* fixed effect design matrix, *c* denotes the number of covariates, including unit vector, population structure (Yu et al., 2006) or principle component (Price et al., 2010), and ***b*** denotes their effect sizes including the intercept *μ*; ***x*** denotes an *n* × 1 genotype vector of the focal QTN, and $\beta ∼N\left(0,\sigma_{g}^{2}\right)$ denotes random QTN effect; the variable ***u*** is a random vector and can be used to account for additional additive effects, such as polygenic effects and other additive confounding factors, $u∼MVN\left(0,K\sigma_{k}^{2}\right)$ is multivariate normal distribution, $\sigma_{k}^{2}$ denotes the variance component of polygenic effects, ***K*** denotes an *n* × *n* genetic relatedness matrix; and $\varepsilon ∼MVN\left(0,\sigma_{e}^{2}I_{n}\right)$ denotes independent and identically distributed noise, $\sigma_{e}^{2}$ is residual error variance, and ***I***_*n*_ is an *n* × *n* identity matrix.

#### Parameter Inference and Score Test for Association Test

For parameter inference, a marginalized form of Model (1) is considered, which is obtained by integrating over the QTN effects β and the polygenic random effect component ***u***

$$y∼N\left(Xb,\underset{QTN}{\underset{︸}{\sigma_{g}^{2}xx^{T}}}+\underset{u}{\underset{︸}{\sigma_{k}^{2}K}}+\underset{noise}{\underset{︸}{\sigma_{e}^{2}I_{n}}}\right)$$ (2)

Note that various methods of inferring a genetic relatedness matrix have been proposed. In this study, we used a marker-inferred genetic relatedness matrix (Price et al., 2010) defined as

$$\begin{array}{l} K=\frac{1}{m}\sum_{i=1}^{m}x_{i}x_{i}^{T}\\ =\frac{1}{m}GG^{T} \end{array}$$ (3)

Here, $G=\left(x_{1},x_{2},\cdots ,x_{m}\right)$ is the whole genotype matrix, *m* is the number of markers. Let $\Sigma =\sigma_{g}^{2}xx^{T}+\sigma_{k}^{2}K+\sigma_{e}^{2}I_{n}$ , and Σ is a positive semi-definite symmetric matrix. Now the multivariate normal distribution is

$$f\left(y\right)=\frac{1}{\sqrt{{\left(2\pi \right)}^{n}\left|\Sigma \right|}}\exp\left\{-\frac{1}{2}{\left(y-Xb\right)}^{T}\Sigma^{-1}\left(y-Xb\right)\right\}$$ (4)

The following log likelihood function can be easily obtained

$$L\left(\theta \right)=-\frac{n}{2}\log\left(2\pi \right)-\frac{1}{2}\log\left|\Sigma \right|-\frac{1}{2}\left\{{\left(y-Xb\right)}^{T}\Sigma^{-1}\left(y-Xb\right)\right\}$$ (5)

where $\theta =\left(\sigma_{g}^{2},\lambda \right)$ , nuisance parameter $\lambda =\left(b,\beta ,\sigma_{k}^{2},\sigma_{e}^{2}\right)$ . We note that the hypothesis for β , $H_{0}:\beta =0,H_{1}:\beta \neq 0$ is equivalent to $H_{0}:\sigma_{g}^{2}=0,H_{1}:\sigma_{g}^{2}>0$ . The score test statistics can be computed analogously to the procedure described in Wu et al. (2011).

$$\begin{array}{l} T_{\mathrm{score}}=\frac{1}{2}{\left(y-X\hat{b}\right)}^{T}M_{0}^{-1}xx^{T}M_{0}^{-1}\left(y-X\hat{b}\right)\\ =\frac{1}{2}y^{T}Pxx^{T}Py\\ =\frac{1}{2}{\left\|x^{T}Py\right\|}^{2} \end{array}$$ (6)

where we have defined

$$P=M_{0}^{-1}-M_{0}^{-1}X{\left(X^{T}M_{0}^{-1}X\right)}^{-1}X^{T}M_{0}^{-1}$$ (7)

In the model in Eq. 6, the vector ***b*** can be estimated *via* null model maximum likelihood estimation (MLE):

$$\hat{b}={\left(X^{T}M_{0}^{-1}X\right)}^{-1}X^{T}M_{0}^{-1}y$$ (8)

The matrix $M_{0}$ denotes the total covariance matrix estimated under the null model

$$M_{0}={\hat{\sigma }}_{k}^{2}K+{\hat{\sigma }}_{e}^{2}I_{n}$$ (9)

where ${\hat{\sigma }}_{k}^{2}$ and ${\hat{\sigma }}_{e}^{2}$ correspond to the null model moment estimation of $\sigma_{k}^{2}$ and $\sigma_{e}^{2}$ (Wu and Sankararaman, 2018). The introduction of the ***K*** matrix makes $M_{0}$ a dense matrix, which presents a significant challenge in computation. However, we adopt preconditioned conjugate gradient iteration to solve this problem (Legarra and Misztal, 2008; VanRaden, 2008), i.e., computation of expressions of the form $M_{0}^{-1}y$ and $\left[M_{0}^{-1}X_{1},\cdots ,M_{0}^{-1}X_{c}\right]$ , which can improve computing speed and reduce memory usage, particularly for large individuals. The statistics *T*_*score*_ follows the chi-square distribution with one degree of freedom

$$T_{\mathrm{score}}∼\chi_{1}^{2}$$ (10)

*p*-values can be computed *via* Davies method (Davies, 1980).

#### Empirical Bayes Estimation for QTN Effects

We conduct variable selection in a multi-locus model

$$y=Xb+\sum_{i=1}^{q}x_{i}\beta_{i}+\varepsilon$$ (11)

where ***y***, ***X***, ***b*** and ***ε*** are the same as those in Model (1); *q* is the number of markers selected in single-locus scanning; *β*_*i*_ is the random effect for marker *i*, and ***x***_*i*_ is the corresponding designed matrix for *β*_*i*_. Obviously, the parameters of interest to be estimated are $\left(\beta_{1},\beta_{2},\cdots \beta_{q}\right)$ .

Empirical Bayes Xu (2010) was performed to estimate the QTN effects in Model (11). In this method, each QTN effect *β*_*i*_ is viewed as random. With the Bayesian hierarchical model, a normal prior is adopted for $\beta_{i}∼N\left(0,\sigma_{i}^{2}\right)$ , and the scaled inverse $\chi^{2}$ prior for $\sigma_{i}^{2}$ , $P\left(\sigma_{i}^{2}|\tau ,\omega \right)\propto {\left(\sigma_{i}^{2}\right)}^{-\frac{1}{2}\left(\tau +2\right)}\exp\left(-\frac{\omega }{2\sigma_{i}^{2}}\right)$ , here, (τ,ω)=(0,0) is used, that is the Jeffrey’s prior (Figueiredo, 2003). The following shows the procedure of Empirical Bayes for parameter estimation.

1) Initial-step: Assign initial values to parameters with

$$\begin{array}{l} b={\left(X^{T}X\right)}^{-1}X^{T}y\\ \sigma_{e}^{2}=\frac{1}{n}{\left(y-Xb\right)}^{T}\left(y-Xb\right)\\ \sigma_{i}^{2}={\left[{\left(x_{i}^{T}x_{i}\right)}^{-1}x_{i}^{T}\left(y-Xb\right)\right]}^{2}+{\left(x_{i}^{T}x_{i}\right)}^{-1}\sigma_{e}^{2} \end{array}$$ (12)

2) Expectation-step: QTN effect can be estimated by

$$E\left(\beta_{i}\right)=\sigma_{i}^{2}x_{i}^{T}\Sigma^{-1}\left(y-Xb\right)$$ (13)

where $\Sigma =\sum_{i=1}^{q}x_{i}x_{i}^{T}\sigma_{i}^{2}+I\sigma_{e}^{2}$ .

3) Maximization-step: Update parameters $b,\;\sigma_{e}^{2},\;\sigma_{i}^{2}$

$$\begin{array}{l} b={\left(X^{T}\Sigma^{-1}X\right)}^{-1}X^{T}\Sigma^{-1}y\\ \sigma_{e}^{2}=\frac{1}{n}{\left(y-Xb\right)}^{T}\left(y-Xb-\sum_{i=1}^{q}x_{i}E(\beta_{i})\right)\\ \sigma_{i}^{2}=\frac{E\left(\beta_{i}^{T}\beta_{i}\right)+\omega }{\tau +3} \end{array}$$ (14)

where $E\left(\beta_{i}^{T}\beta_{i}\right)=E\left(\beta_{i}^{T}\right)E\left(\beta_{i}\right)+tr\left[\mathrm{var}\left(\beta_{i}\right)\right]$ , $\mathrm{var}\left(\beta_{i}\right)=I\sigma_{i}^{2}-\sigma_{i}^{2}x_{i}^{T}\Sigma^{-1}x_{i}\sigma_{i}^{2}$ and (τ,ω)=(0,0) .

Repeat 2) and 3) until convergence. All the markers with $\left|{\hat{\beta }}_{i}\right|\leq 10^{-4}$ were excluded in the first step, the likelihood ratio test was then conducted on the estimate of other marker effect *β*_*i*_. Because Empirical Bayes is a multi-locus model, there is no requirement for Bonferroni correction (Wang et al., 2016). Instead of using 0.05/*m* as a significant threshold, where *m* is the number of markers, the criterion of logarithm of the odds (LOD) = 3.0 was set up (Wang et al., 2016; Ren et al., 2018; Wen et al., 2018). This criterion is frequently adopted in linkage analysis and is the equivalent of $P=\mathrm{Pr}\left(\chi_{1}^{2}>3.0\times 4.605\right)\approx 0.0002$ , where LOD follows a $\chi_{1}^{2}$ distribution and LOD = LR/4.605.

### Simulation Study

We performed three simulation experiments to validate ScoreEB. In the first simulation experiment, 216,130 SNPs in Atwell et al. (2010) was used as the simulated genotype. The sample size was equal to the number of individuals, that was 199. Six QTNs were simulated and placed on the SNPs with allelic frequencies of 0.30, their heritability was set as 0.10, 0.15, 0.05, 0.05, 0.05, and 0.05, and their positions and effects are listed in Table 1. Three level of heritability (0.05, 0.10 and 0.15) was to investigate the ability of different methods to detect QTNs with different heritability. The differences between our simulation study and previous methods mrMLM (Wang et al., 2016) and ISIS EM-BLASSO (Tamba et al., 2017) were as follows: 1) We used 216,130 SNPs as the simulated genotype rather than employed 10,000 SNP genotypes. If the computational capacity allowed, more SNP markers could reflect the reality. 2). The genotype coding was different. We used 0, 1 and 2 to represent “aa”, “Aa” and “AA”, however, they used −1, 0 and 1 to represent “aa”, “Aa” and “AA”. 3). The order of QTNs was sorted based upon the heritability of 0.10, 015, 0.05, 0.05, 0.05 and 0.05 in our study. The SNPs in high LD (linkage disequilibrium) with the assumed QTNs are listed in Table 2. The phenotype including a polygenic background was simulated by the model $y=\mu +\sum_{i=1}^{6}x_{i}\beta_{i}+u+\varepsilon$ , where $u∼MVN\left(0,\sigma_{k}^{2}\times K\right)$ is the polygenic effect and $\varepsilon ∼MVN\left(0,\sigma_{e}^{2}I_{n}\right)$ is the residual error. The mean value of the phenotype μ was set to 10.0. Here we set residual variance $\sigma_{e}^{2}=10.0$ and polygenic variance $\sigma_{k}^{2}=2.0$ . With $h_{t}^{2}=\sigma_{g}^{2}/\left(\sigma_{g}^{2}+\sigma_{e}^{2}+\sigma_{k}^{2}\right)=0.05\times 4+0.10+0.15=0.45$ , that is $\sigma_{g}^{2}/\left(\sigma_{g}^{2}+10+2\right)=0.45$ , total genetic variance $\sigma_{g}^{2}$ and each QTN genetic variance $\sigma_{i}^{2}\left(i=1,\cdots ,6\right)$ could be obtained. The heritability of polygenic effect $h_{k}^{2}$ is $\sigma_{k}^{2}/\left(\sigma_{g}^{2}+\sigma_{e}^{2}+\sigma_{k}^{2}\right)=2/\left(9.82+10+2\right)\approx 0.092$ , which is nearly one QTN with heritability 0.10, this can make polygenic effect having a moderate impact. Each QTN true effect can be obtained from $\;\beta_{i}=\sqrt{\left[h_{t}^{2}\left(\sigma_{e}^{2}+\sigma_{k}^{2}\right)/\left(1-h_{t}^{2}\right)+\sigma_{e}^{2}+\sigma_{k}^{2}\right]h_{i}^{2}/\left[4\eta_{i}\left(1-\eta_{i}\right)\right]}$ , where $\eta_{i}$ denotes the minor allele frequency (MAF), and $h_{i}^{2}$ denotes the heritability of each QTN. The simulation experiment was repeated 1,000 times. The false positive rate (FPR) was defined as the ratio between the number of non QTNs wrongly categorized as positive and the total number of actual non QTNs. To evaluate the variance and bias of each QTN effect estimate, the mean squared error (MSE) was calculated. We defined MSE as

$$MSE_{i}=\frac{1}{R_{i}}{\sum_{s}^{R}\left({\hat{\beta }}_{is}-\beta_{i}\right)}^{2}$$ (15)

where $R_{i}$ is the total number of detected *i*th QTN, i=1,⋯,6 is the *i*th QTN, ${\hat{\beta }}_{is}$ is the estimated effect of QTN *i* from the *s*th repeat, and $\beta_{i}$ is the true effect of QTN *i*.

**TABLE 1:** Comparison of mean squared errors (MSE) for each QTN among ScoreEB, mrMLM, FASTmrEMMA, ISIS EM-BLASSO, HRePML and GEMMA methods in the first simulation study^a^.

QTN	Chr.	Pos. (bp)	*R* ^2^	Effect	Mean squared errors (MSE)					
					ScoreEB	mrMLM	FASTmrEMMA	ISIS EM-BLASSO	HRePML	GEMMA
1	1	11,298,364	0.10	1.6171	0.1000	0.1064	0.5052	0.1258	0.2841	0.3925
2	2	5,134,228	0.15	1.9806	0.2108	0.1929	0.3803	0.2046	0.6025	0.2303
3	2	5,066,968	0.05	1.1435	0.0793	0.1051	0.4022	0.0838	0.0871	10.4514
4	2	5,464,675	0.05	1.1435	0.0721	0.1024	0.6352	0.0886	0.0524	11.2228
5	2	6,137,189	0.05	1.1435	0.0690	0.0728	0.2705	0.0828	0.0942	0.7748
6	1	11,655,607	0.05	1.1435	0.0692	0.0652	0.2568	0.0940	0.1128	10.8202
Average MSE					0.1001	0.1075	0.4084	0.1132	0.2055	5.6487

a In the first simulation study, the dataset consists of 199 individuals and 216,130 single nucleotide polymorphism (SNP) markers with 1,000 replicates. Six true QTNs are set in each replicate.

**TABLE 2:** The SNPs in high LD (linkage disequilibrium) with the assumed QTNs in the first simulation study^a^.

QTN	Chr.	Position	SNP	Chr.	Position	r-square	D’
3	2	5,066,968	S2_5063677	2	5,063,677	0.6975	0.9477
4	2	5,464,675	S2_5457514	2	5,457,514	0.5145	0.7520
			S2_5459900	2	5,459,900	0.5801	0.7800
			S2_5460143	2	5,460,143	0.6309	0.8039
			S2_5465839	2	5,465,839	0.5443	0.7556
			S2_5467272	2	5,467,272	0.5964	0.7816
			S2_5468325	2	5,468,325	0.5762	0.7591
			S2_5468547	2	5,468,547	0.5198	0.7735
			S2_5470625	2	5,470,625	0.5563	0.7549
			S2_5470963	2	5,470,963	0.5600	0.7573
6	1	11,655,607	S1_11655586	1	11,655,586	0.8158	0.9732
			S1_11655834	1	11,655,834	0.6452	0.9726
			S1_11657017	1	11,657,017	0.5327	0.8651
			S1_11657744	1	11,657,744	0.5799	0.8929

a The LD statistics r-square and D’ are obtained by PLINK v1.90, and the SNP is regarded as in high LD with the assumed QTNs when r-square is greater than 0.5. The r-squares between SNPs and QTN 1, 2 and 5 are no greater than 0.5.

In the second simulation experiment, the phenotypes without polygenic effect were simulated by the model $y=\mu +\sum_{i=1}^{6}x_{i}\beta_{i}+\varepsilon$ , where $\varepsilon ∼MVN\left(0,\sigma_{e}^{2}\times I_{n}\right)$ . Other parameters were the same as those in the first experiment, and all the parameters are listed in Supplementary Table S1.

In the third simulation experiment, we intended to investigate the influence of the sample size on the running time. The sample size was set to 200, 500, 1,000, and 2,000, respectively. Meanwhile, the number of markers was fixed at 50,000. And repetition times was set to 100. In addition, five-fold cross-validation test was performed to decide the choice of two hyperparameters in Empirical Bayes step of ScoreEB at different sample sizes (Supplementary Table S2).

### Real Datasets

We use previously published datasets from multiple species that includes *Arabidopsis thaliana*, rice, maize, cattle and pig.

The *Arabidopsis thaliana* dataset consists of 199 accessions each with 216,130 genotyped SNPs (Atwell et al., 2010), and the phenotype FRI gene expression levels (FRI) is re-analyzed. The rice dataset is conducted analysis based on 44,100 genotyped SNPs across 413 diverse accessions (Zhao et al., 2011). The phenotype 2007 year flowering time at Arkansas is used to be analyzed. The maize genotype dataset consists of 2,279 inbred lines, each with 681,258 SNPs. The phenotype is flowering time measured as days to silk (Romay et al., 2013). The cattle dataset has 5,254 samples with 42,551 genotyped SNPs. The phenotype is milk yield (mkg), which is an important economic trait (Zhang et al., 2015). The pig dataset consists of 4,260 samples each with 47,157 genotyped SNPs, and all SNP markers were mapped to *Sus scrofa* genome build 11.1. The growth performance related phenotype AGE (days to 100 kg) is re-analyzed (Ramos et al., 2009; Tang et al., 2019). The SNPs with a minor allele frequency (MAF) of 5% or less are filtered out. And the SNPs with missing rate of 20% or more are deleted.

## Results

To validate the performance of ScoreEB, three simulation experiments and five real datasets analysis were carried out. Each experiment was analyzed by six methods: a fast score test integrated with Empirical Bayes (ScoreEB), multi-locus random-SNP-effect mixed linear model (mrMLM), fast multi-locus random-SNP-effect EMMA (FASTmrEMMA), iterative modified-sure independence screening EM-Bayesian lasso (ISIS EM-BLASSO), hybrid of restricted and penalized maximum likelihood (HRePML) and genome-wide efficient mixed model association (GEMMA). We performed simulated and real data analysis using six GWAS methods on the same computer (Intel^®^ Core™ i9-10855H CPU 2.40 GHz, Memory 64 GB), which has 8 cores and 16 threads. The versions of R and gcc are r-base-4.0.5 and 7.5.0, respectively, based on the Ubuntu 18.04 operating system.

### Simulation Study

#### Statistical Power Under Different Levels of FDR and Type I Error

**FIGURE 1:**
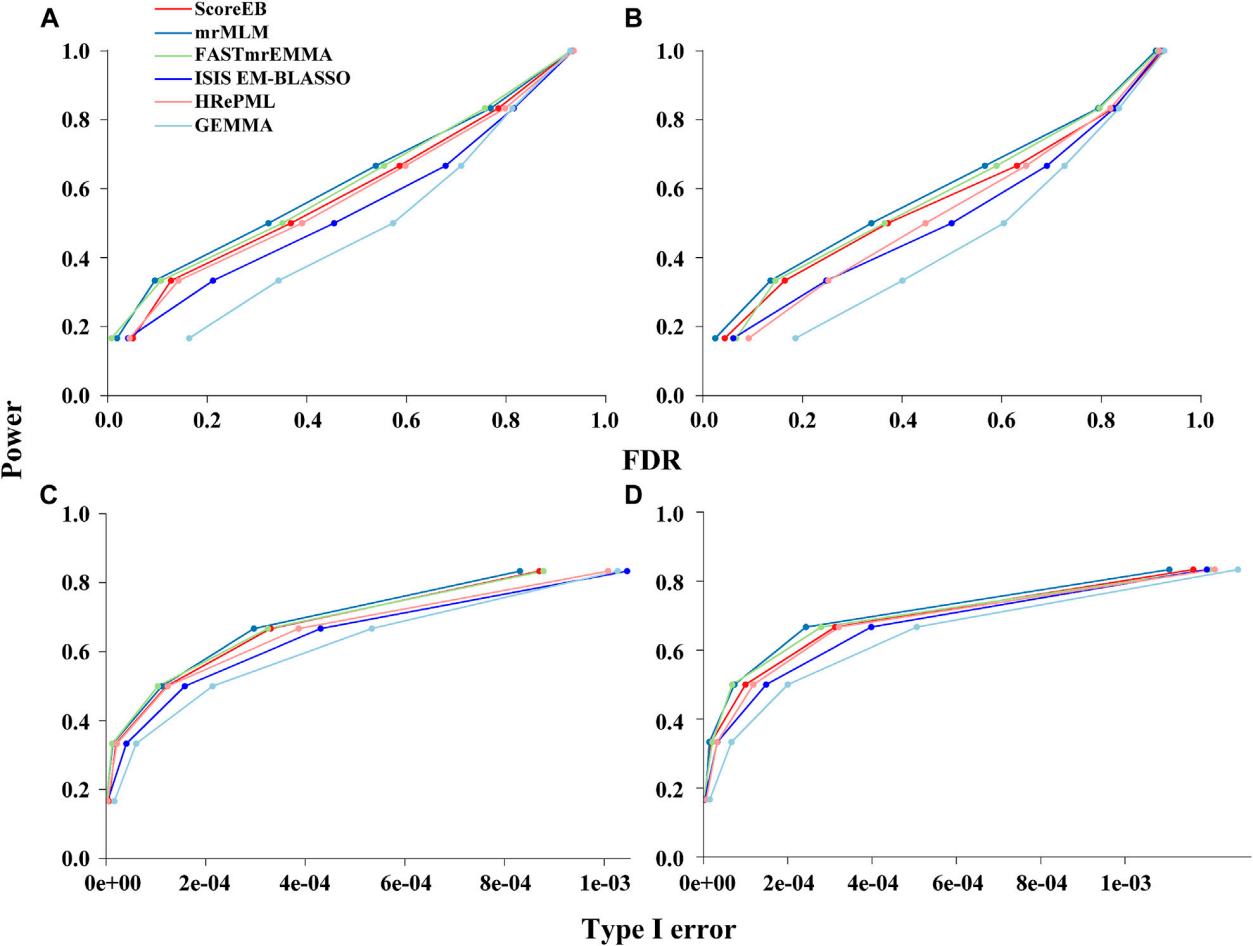
Performances of six methods (ScoreEB: a fast score test integrated with Empirical Bayes, mrMLM: multi-locus random-SNP-effect mixed linear model, FASTmrEMMA: multi-locus random-SNP-effect EMMA, ISIS EM-BLASSO: iterative modified-sure independence screening EM-Bayesian lasso, HRePML: hybrid of restricted and penalized maximum likelihood and GEMMA: genome-wide efficient mixed model association) using simulation data. Statistical power under different levels of FDR in simulation 1 **(A)** and simulation 2 **(B)**, and statistical power under different levels of Type I error in simulation 1 **(C)** and simulation 2 **(D).**

Genetic markers were classified into the ones on QTN-area and non-QTN area to evaluate statistical power under different levels of FDR and Type I error. And 10^3^ bp was selected as the window size in our simulation analysis. In the first simulation experiment where six QTN effects and an additive polygenic effect were involved, the area under the Power-FDR curve (AUC.FDR) for ScoreEB, mrMLM, FASTmrEMMA, ISIS EM-BLASSO, HRePML and GEMMA methods were 0.4405, 0.4651, 0.4583, 0.4020, 0.4385 and 0.3358, respectively, showing that ScoreEB along with mrMLM and FASTmrEMMA has the similar power, which are significantly higher than GEMMA (Figure 1A). The power of HRePML and ISIS EM-BLASSO were lower than ScoreEB, while higher than GEMMA. In the second simulation experiment when only six QTN effects were added to the phenotype, the AUC.FDR for the above six methods were 0.4241, 0.4498, 0.4354, 0.3743, 0.3883 and 0.3129, respectively, indicating that the three multi-locus methods ScoreEB, mrMLM and FASTmrMLM still have the higher power than other methods, especially the single-locus method GEMMA (Figure 1B). And the area under the Power-Type I error curve demonstrated the similar trends (Figures 1C,D). Clearly, the power of ScoreEB is comparable to that of the other two multi-locus methods mrMLM and FASTmrEMMA.

#### Accuracy for Estimated QTN Effects

**FIGURE 2:**
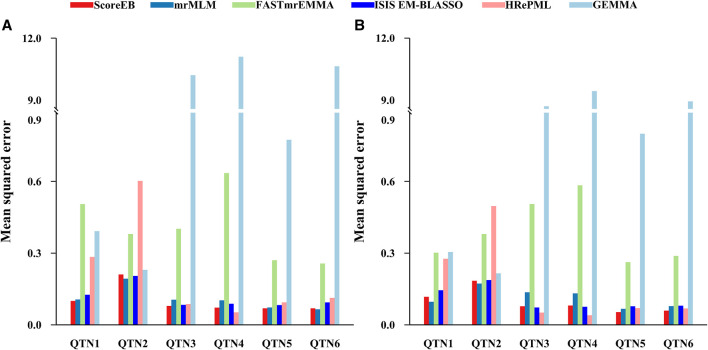
Comparison of mean squared error (MSE) of six simulated QTNs using six methods (ScoreEB, mrMLM, FASTmrEMMA, ISIS EM-BLASSO, HRePML and GEMMA) in simulation 1 **(A)** and simulation 2 **(B)**.

We used the mean squared error (MSE) to measure the accuracy of QTN effect estimation. The smaller the MSE, the better the accuracy of the method. We evaluated the accuracies for all of the six simulated QTNs across six methods. In the first simulation experiment, results demonstrated that the average MSEs with ScoreEB, mrMLM, FASTmrEMMA, ISIS EM-BLASSO, HRePML and GEMMA were 0.1001, 0.1075, 0.4084, 0.1132, 0.2055 and 5.6487, respectively (Table 1 and Figure 2A). The average MSE of ScoreEB is the minimum. Compared with the average MSE of GEMMA, that of ScoreEB is significantly lower. In the second simulation experiment, results showed the same trend, and the average MSEs of the six methods were 0.0955, 0.1137, 0.3871, 0.1064, 0.1674 and 4.9340, respectively (Supplementary Table S1 and Figure 2B). These results indicate that ScoreEB along with mrMLM and ISIS EM-BLASSO has significantly higher accuracy of QTN effect estimation than the single-locus method GEMMA.

### Application to Real Data in *Arabidopsis*, Rice, Maize, Cattle and Pig

**FIGURE 3:**
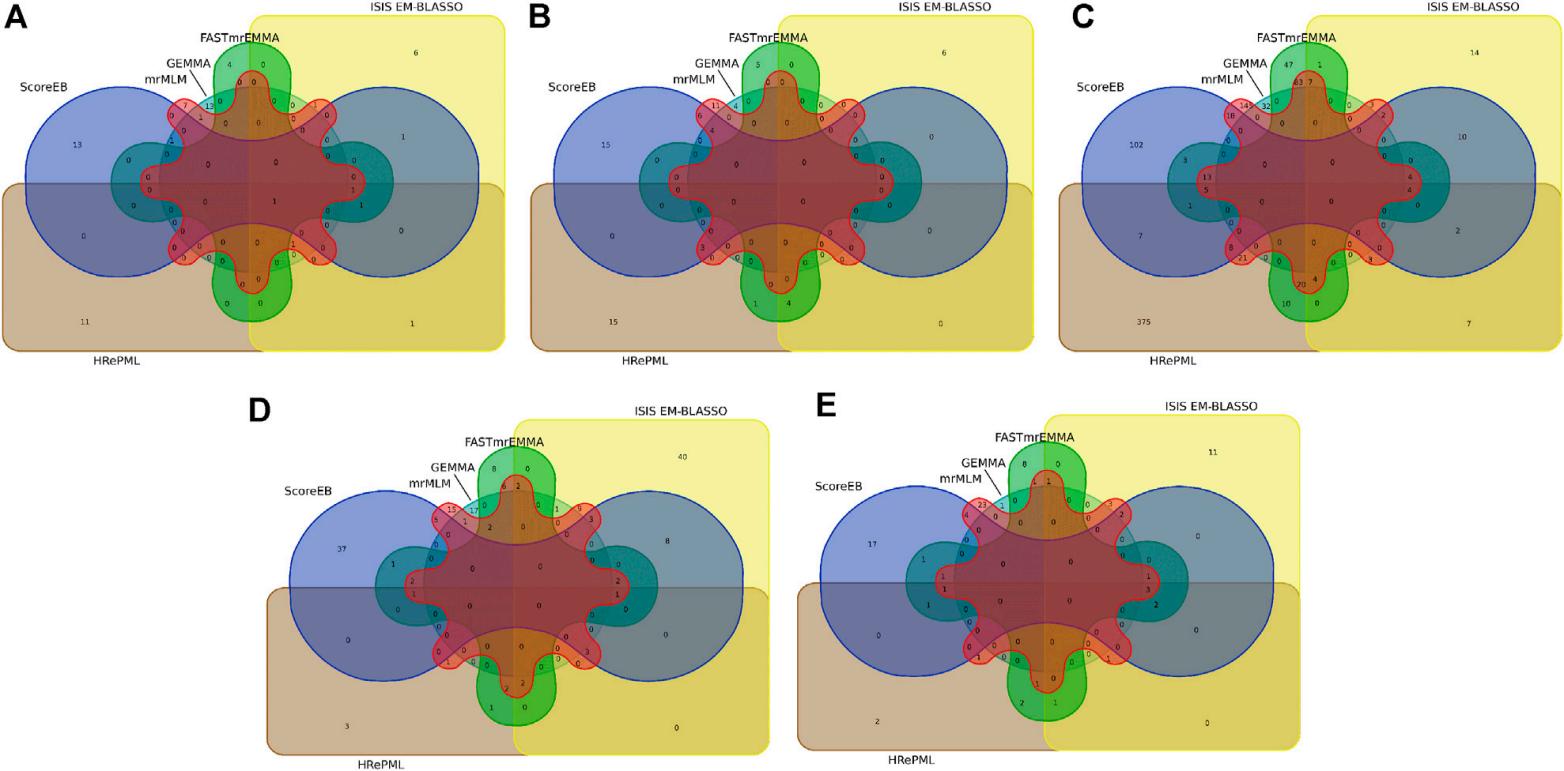
Venn diagram of nearby candidate genes or QTLs identified by six methods (ScoreEB, mrMLM, FASTmrEMMA, ISIS EM-BLASSO, HRePML and GEMMA) on quantitative traits in *Arabidopsis* **(A)**, rice **(B)**, maize **(C),** cattle **(D)** and pig **(E).**

The *Arabidopsis* data set consists of 199 accessions each with 216,130 genotyped SNPs (Atwell et al., 2010). We re-analyzed flowering time related trait FRI of the *Arabidopsis* data by ScoreEB, mrMLM, FASTmrEMMA, ISIS EM-BLASSO, HRePML and GEMMA. These methods identified 8, 3, 3, 8, 10 and 33 SNPs significantly associated with FRI trait, respectively. We then detected previously reported genes associated with these SNPs *via* the *Arabidopsis* website. As a result, 18, 12, 7, 13, 16 and 17 genes were identified by the above six methods respectively, indicating that ScoreEB detected the most previously reported genes. Notably, *FLA* was detected by all the six methods at the same time (Table 3, Supplementary Table S3 and Figure 3A). Previous studies have shown that *FLA* encodes a major determinant of natural variation in *Arabidopsis* flowering time. And dominant alleles of *FLA* confer a vernalization requirement causing plants to overwinter vegetatively. Although GEMMA detected the most SNPs, these SNPs were only associated with 17 genes. Clearly, ScoreEB was more powerful to mine candidate genes than the other methods in analysis of *Arabidopsis*.

**TABLE 3:** Top five associated SNPs identified by ScoreEB on quantitative traits in *Arabidopsis*, rice, maize, cattle and pig.

Species	SNP_ID	Chr.	Position	Effect	Lod	*p* value	Nearby candidate genes or QTLs (base pairs, start: end)
*Arabidopsis*	S4_308466	4	308,466	0.332	11.60	2.70 × 10^−13^	*AHDP* (299,359: 304,508)
	S4_268990	4	268,990	0.227	8.26	6.94 × 10^−10^	*FLA* (269,026: 271,503)
	S1_16446253	1	16,446,253	0.149	7.16	9.35 × 10^−9^	*ATTLP5* (16,439,435: 16,441,844)
	S3_10280193	3	10,280,193	−0.086	4.99	1.64 × 10^−6^	*MER3* (10,273,801: 10,280,362)
	S1_16394129	1	16,394,129	0.093	4.53	4.94 × 10^−6^	*ATY2* (16,398,099: 16,399,878)
Rice	S3_35314180	3	35,314,180	0.043	9.60	2.95 × 10^−11^	*RLCK122* (35,312,451: 35,317,359)
	S7_18408767	7	18,408,767	0.039	5.72	2.86 × 10^−7^	*OsSTA195* (18,393,745: 18,399,059)
	S1_22493100	1	22,493,100	0.024	3.79	2.94 × 10^−5^	*ACO7* (22,489,496: 22,491,483)
	S1_23314458	1	23,314,458	−0.027	3.46	6.56 × 10^−5^	*THI27* (23,311,171: 23,312,581)
	S1_34082456	1	34,082,456	−0.025	3.22	1.18 × 10^−4^	*CYP94D12* (34,084,757: 34,086,514)
Maize	S10_6375466	10	6,375,466	−0.029	9.26	6.57 × 10^−11^	*GRMZM2G052499* (6,357,257: 6,359,007)
	S3_214713620	3	214,713,620	0.008	9.06	1.05 × 10^−10^	*GRMZM2G037644* (214,735,031: 214,738,322)
	S9_12878270	9	12,878,270	−0.014	8.48	4.13 × 10^−10^	*GRMZM2G024530* (12,903,174: 12,908,621)
	S9_123409245	9	123,409,245	0.012	7.97	1.38 × 10^−9^	*GRMZM2G363649* (123,429,468: 123,435,166)
	S4_173930289	4	173,930,289	−0.017	7.59	3.38 × 10^−9^	*GRMZM2G344967* (173,909,713: 173,929,961)
Cattle	S14_1610986	14	1,610,986	−0.542	241.03	2.30 × 10^−243^	*VPS28* (1,693,641: 1,698,490)
	S9_66164662	9	66,164,662	−0.124	16.31	4.50 × 10^−18^	*MRAP2* (66,223,880: 66,287,509)
	S19_22081512	19	22,081,512	−0.097	10.81	1.72 × 10^−12^	*TUSC5* (22,167,563: 22,186,258)
	S1_136656873	1	136,656,873	0.124	10.38	4.72 × 10^−12^	*PPP2R3A* (134,223,427: 134,394,973)
	S6_85505724	6	85,505,724	0.096	10.16	7.88 × 10^−12^	*TMPRSS11F* (85,473,854: 85,512,994)
Pig	WU_10.2_1_179575045	1	161,987,727	−1.701	13.41	3.90 × 10^−15^	*MALT1* (162,076,951: 162,144,880)
	ASGA0089196	1	57,487,161	1.424	8.44	4.59 × 10^−10^	*ANKRD6* (57,438,000: 57,645,958)
	WU_10.2_8_3769689	8	3,371,469	1.254	6.06	1.28 × 10^−7^	*SORCS2* (3,207,098: 3,760,393)
	WU_10.2_13_26791609	13	24,433,904	−1.647	5.84	2.15 × 10^−7^	*MYRIP* (24,347,660: 24,556,735)
	WU_10.2_9_43903117	9	39,084,510	−1.172	5.60	3.80 × 10^−7^	*POU2AF1* (39,119,012: 39,144,606)

With above six methods, we conducted a genome-wide association study based on genotyped 44,100 SNPs across 413 diverse accessions in 2007 year flowering time at Arkansas of rice data (Zhao et al., 2011). The SNPs significantly associated with flowering time for ScoreEB, mrMLM, FASTmrEMMA, ISIS EM-BLASSO, HRePML and GEMMA methods were 8, 7, 3, 3, 8 and 3, respectively. *Via* analysis of gene ontology annotations, the above six methods detected 28, 24, 10, 10, 23 and 8 associated genes, respectively. There were 4 genes located on chromosome 2 identified by ScoreEB, mrMLM and GEMMA three methods at the same time, and 3 genes located chromosome 1 identified by ScoreEB, mrMLM and HRePML simultaneously (Table 3, Supplementary Table S3 and Figure 3B). The results demonstrated that ScoreEB not only detected the most SNPs and associated genes, but also was well consistently with other methods.

The maize flowering time measured as days to silk was re-analyzed with the same six methods. The genotype of maize data consists of 2,279 inbred lines, each with 681,258 SNPs (Romay et al., 2013). The number of significantly associated SNPs detected by these methods was 284, 606, 343, 98, 868 and 79, respectively. And the number of identified genes or QTLs around these SNPs was 179, 340, 202, 61, 467 and 32, respectively. Results indicated that the HRePML detected the most genes or QTLs, followed by mrMLM, FASTmrEMMA, ScoreEB, ISIS EM-BLASSO and GEMMA. And the number of genes detected by GEMMA and ISIS EM-BLASSO was far less than that of other four multi-locus methods. We counted the top five associated SNPs identified by ScoreEB, the Lod values of which ranged from 7.59 to 9.26. And nearby these SNPs, maize flowering time genes were found, such as, *GRMZM2G052499*, *GRMZM2G037644 etc* (Table 3). There were 17 genes or QTLs identified at least by four methods simultaneously (Supplementary Table S3 and Figure 3C). Results showed that ScoreEB was also comparable to HRePML, mrMLM and FASTmrEMMA methods in analysis of maize.

In addition to its application to flowering time related traits in plants, we analyzed the quantitative traits of cattle and pig. The cattle data set consists of 5,254 samples each with 42,551 genotyped SNPs (Zhang et al., 2015). In the analysis of milk yield (mkg), ISIS EM-BLASSO and ScoreEB detected the most number of significantly associated SNPs, which were 103 and 90, respectively. And there were 72, 34, 17 and 22 significantly associated SNPs identified by mrMLM, FASTmrEMMA, HRePML and GEMMA. *Via* analysis of gene ontology annotations, the above six methods detected 71, 63, 57, 30, 14 and 21 associated genes, respectively. It was worth noting that ScoreEB identified the *VPS28* gene, which was extremely significant with 241.03 lod value and 2.30 × 10^−243^ *p* value (Table 3). The *VPS28* gene could regulate milk fat synthesis through modulating the ubiquitination-lysosome and ubiquitination-proteasome systems (Liu et al., 2018). Besides *VPS28* gene, there was other 8 genes identified by at least four methods simultaneously (Supplementary Table S3 and Figure 3D). These results supported that ScoreEB was effective in cattle application.

Using ScoreEB, mrMLM, FASTmrEMMA, ISIS EM-BLASSO, HRePML and GEMMA six methods, we re-analyzed AGE trait in pig based on 47,157 genotyped SNPs 4,260 samples (Ramos et al., 2009; Tang et al., 2019). The number of significantly associated SNPs detected by these methods was 50, 57, 28, 33, 16 and 1, respectively, and the number of identified genes or QTLs around these SNPs was 33, 43, 24, 25, 15 and 1, respectively. There were 6 genes identified at least by four methods simultaneously and ScoreEB detected all these 6 genes, such as, *ANKRD6*, *MALT1 etc*., (Table 3, Supplementary Table S3 and Figure 3E). Meanwhile, ScoreEB and mrMLM identified the most number of associated genes. The single-locus method GEMMA had a poor performance with only 1 gene identified. Results demonstrated ScoreEB was also powerful to mine candidate genes in pig.

### Computational Efficiency

#### Time Complexity

We compared time complexity over M markers and N individuals among the above six methods. In ScoreEB, the time complexity of first stage is *O*(MN), and that of the second stage is *O*(tqN^2^), here, t is the number of iterations required for expectation-maximization (EM) method to converge, q is the number of markers selected in the first stage, and t, q is much smaller than M. The time complexity of mrMLM and FASTmrEMMA in the first stage is difficult to make sure because they call other complicated algorithms, and that of ISIS EM-BLASSO is mainly limit to iterative modified-sure independence screening (ISIS) step, however, the time complexity of these three methods in the second stage are the same with that of ScoreEB. The time complexity of HRePML is greatly affected by limited memory Broyden-Fletcher-Goldfarb-Shanno (BFGS) method. And the time complexity of GEMMA is *O*(MN^2^). The multi-locus method ScoreEB along with mrMLM, FASTmrEMMA and ISIS EM-BLASSO is constrained by Empirical Bayes in the second step, ScoreEB has a high computational efficiency when the number of individuals is not very large (*n* < 3,000).

#### Observed Running Time

**FIGURE 4:**
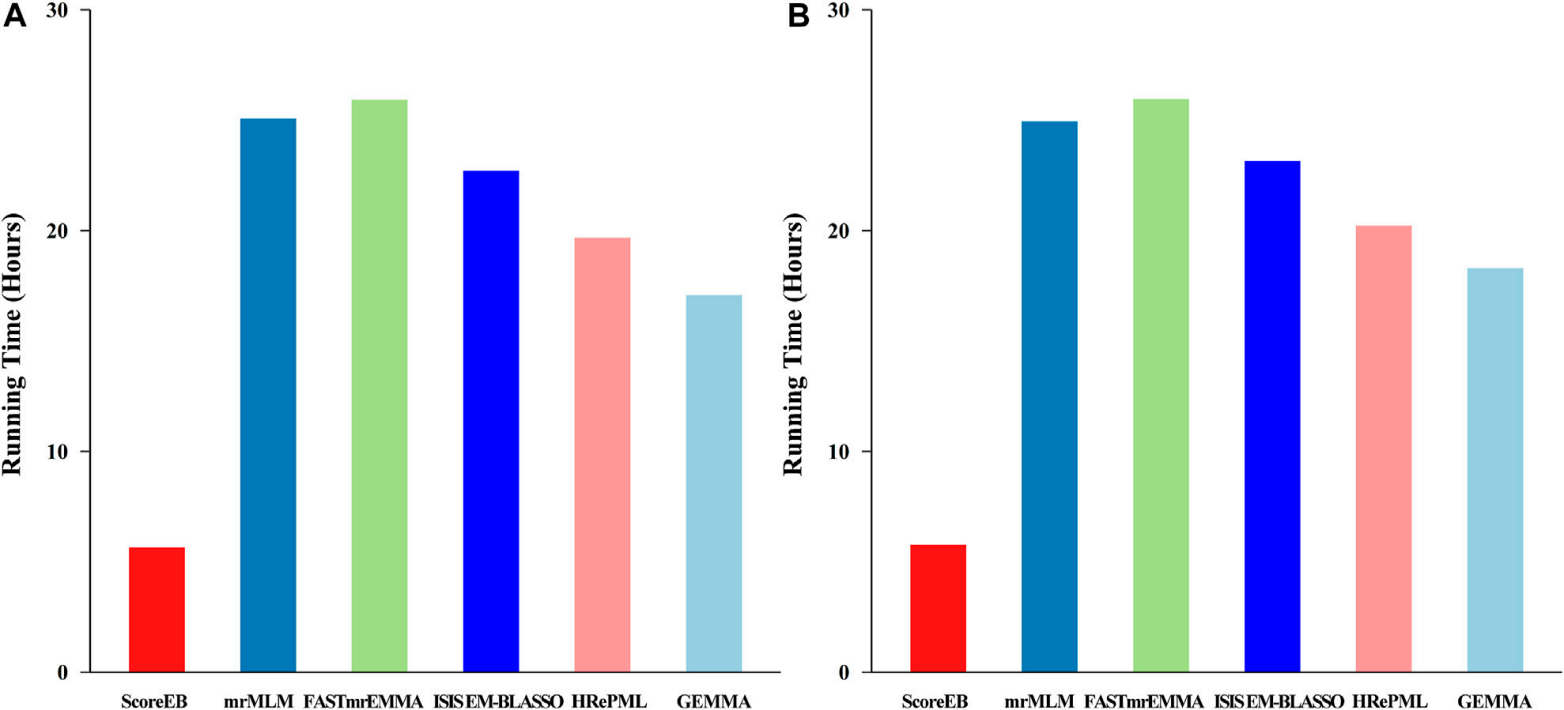
Comparison of 1,000 replicates running time using six methods (ScoreEB, mrMLM, FASTmrEMMA, ISIS EM-BLASSO, HRePML and GEMMA) in simulation 1 **(A)** and simulation 2 **(B)**.

In the first simulation experiment, the dataset consists of 199 individuals and 216,130 single nucleotide polymorphism (SNP) markers with 1,000 replicates. The total running time for ScoreEB, mrMLM, FASTmrEMMA, ISIS EM-BLASSO, HRePML and GEMMA methods were 5.6419, 25.0795, 25.9247, 22.7102, 19.6781 and 17.0846 h, respectively (Figure 4A). The ScoreEB is the most fast, followed by GEMMA, HRePML, ISIS EM-BLASSO, mrMLM and FASTmrEMMA. Clearly, ScoreEB was about 4 times faster than mrMLM and FASTmrEMMA. However, GEMMA was the second faster at the expense of statistical power and estimating QTN effects. In the second simulation experiment, running time shows a similar trend. ScoreEB only take 5.7727 h, which are significantly faster than mrMLM, FASTmrEMMA, ISIS EM-BLASSO, HRePML and GEMMA with 24.9507, 25.9596, 23.1574, 20.2281 and 18.2995 h, respectively (Figure 4B). Results demonstrate that ScoreEB improves computing efficiency considerably compared with the other five methods.

**FIGURE 5:**
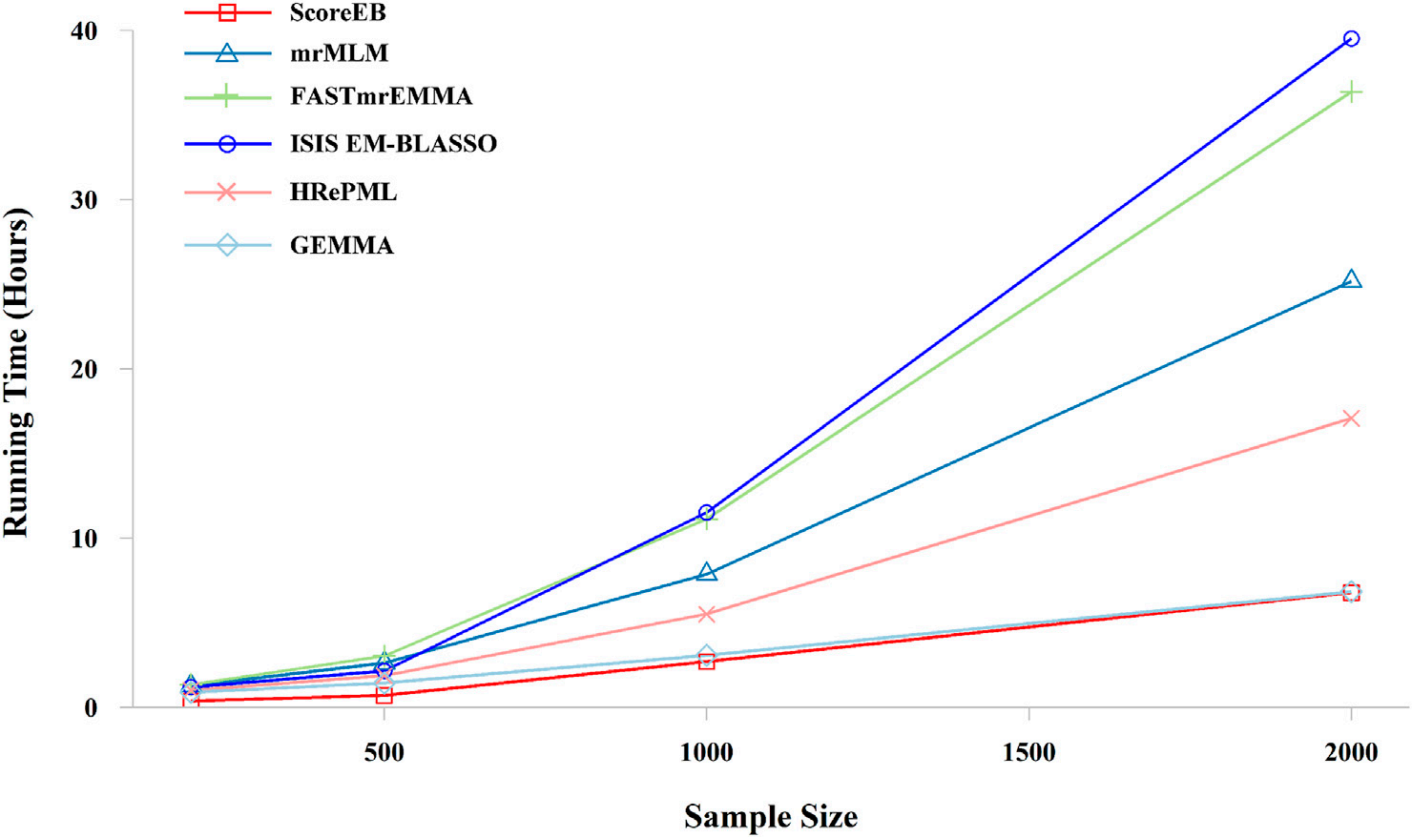
Effect of the sample size on running time with six methods (ScoreEB, mrMLM, FASTmrEMMA, ISIS EM-BLASSO, HRePML and GEMMA) in simulation 3.

In the third simulation experiment, the dataset consists of 50,000 markers with 200, 500, 1,000 and 2,000 samples, respectively. And repetition times was set to 100. ScoreEB is always the most fast with 0.3708, 0.7041, 2.6965 and 6.7638 h at different sample size, and GEMMA and HRePML are always the second and the third fast, respectively (Table 4 and Figure 5). With sample size 200 and 500, ScoreEB is much faster than GEMMA and HRePML, and the order of computational efficiency in other three methods is ISIS EM-BLASSO, mrMLM and FASTmrEMMA. At these two sample sizes, FASTmrEMMA is the slowest. When the sample size increases to 1,000 and 2,000, the advantage of ScoreEB over GEMMA in computing speed is becoming less and less. The main reason is that Empirical Bayes is relatively slow to calculate large samples. However, ScoreEB is still much faster than mrMLM, FASTmrEMMA and ISIS EM-BLASSO, although the second step of these four methods are using the same Empirical Bayes. The speed improved of ScoreEB is mainly due to the use of score test and preconditioned conjugate gradient in the first step. At sample size of 1,000 and 2,000, ISIS EM-BLASSO is the slowest with 11.5060 and 39.5193 h, rather than FASTmrEMMA again (Table 4 and Figure 5). The possible reason is that the number of markers retained in the initial screening of ISIS EM-BLASSO is more than that of FASTmrEMMA. In summary, ScoreEB and GEMMA have considerable advantage in computational efficiency.

**TABLE 4:** Comparison of running time at different sample size with ScoreEB, mrMLM, FASTmrEMMA, ISIS EM-BLASSO, HRePML and GEMMA methods^a^.

Sample size	Running time (hours)					
	ScoreEB	mrMLM	FASTmrEMMA	ISIS EM-BLASSO	HRePML	GEMMA
200	0.3708	1.2785	1.3456	1.1938	1.0061	0.8861
500	0.7041	2.6229	3.0340	2.1576	1.8669	1.4361
1,000	2.6965	7.8627	11.1218	11.5060	5.5006	3.0722
2,000	6.7638	25.1743	36.3763	39.5193	17.0708	6.8278

a The number of markers is set to 50,000, and running time is the total hours of 100 replicates at each sample size.

## Discussion

We have shown that the new method ScoreEB can significantly improve computational efficiency by combining the score test and Empirical Bayes within the linear mixed model, compared with popular methods mrMLM (Wang et al., 2016), FASTmrEMMA (Wen et al., 2018), ISIS EM-BLASSO (Tamba et al., 2017), HRePML (Ren et al., 2020) and GEMMA(Zhou and Stephens, 2012). In the application of ScoreEB to simulation studies, the proposed approach consistently recorded the higher power. More importantly, it also remained estimation accuracy of QTN effects and effectively controlled the false positive rate (Table 1, Supplementary Table S1 and Figures 1, 2). Analysis of real *Arabidopsis,* rice, maize, cattle and pig data, confirmed the effectiveness of ScoreEB, which identified the most candidate genes (Table 3, Supplementary Table S3 and Figure 3).

With the rapid growth of genomic data, computational efficiency has become a popular research issue. Existing multi-locus GWAS methods, such as, mrMLM (Wang et al., 2016), pKWmEB (Ren et al., 2018), FASTmrEMMA (Wen et al., 2018) and MLMM (Segura et al., 2012) are all considerably slower than the single-locus method GEMMA. As described in pKWmEB paper, pKWmEB is about 21 times slower than GEMMA, and mrMLM is about 8 times slower than GEMMA. This is an important motivation for developing the multi-locus method ScoreEB. In contrast to the mrMLM method, we adopt a fast score test in initial single-locus scanning, rather than wald test. In the initial screening, we focus on the significant QTN, rather than the estimation of QTN effects, hence, the score test is a more appropriate choice. The score test only requires maximum likelihood estimation (MLE)under the null model (Song et al., 2018). Simulation studies show that ScoreEB is significantly faster than mrMLM, FASTmrEMMA, ISIS EM-BLASSO, HRePML and GEMMA (Table 4 and Figures 4, 5). And HRePML is faster than mrMLM, FASTmrEMMA and ISIS EM-BLASSO, one possible reason is that HRePML is programmed by C++ language, while other methods are developed using R language. Although the runs of the single-locus method GEMMA were slightly faster than the other multi-locus methods, its statistical power was considerably lower than that of other multi-locus methods, as a result of requiring a Bonferroni correction for multiple tests. The significance level for single-locus test is always adjusted by 0.05/*m*, where *m* is the number of markers. If multiple tests are not used in single-locus scanning to improve power, the significance level is often difficult to determine, and an inappropriate significant level will increase the false positive rate. ScoreEB provides a good solution to this problem by applying Empirical Bayes in a multi-locus model. LOD = 3.0 is set as the significance level, which is widely used in other multi-locus methods (Wang et al., 2016; Ren et al., 2018; Wen et al., 2018). In addition, the new method, ScoreEB, demonstrates accurate estimation of QTN effects, which compensates for a shortcoming of the simple single-locus score test.

The Empirical Bayes model (Xu, 2010) is one core step on inferring the QTN effects in ScoreEB. We have noticed that the hyperparameters could affect the estimates of QTN effects. To determine the best choice of two hyperparameters (τ,ω) , five-fold cross-validation test was performed at sample size 200, 500, 1,000 and 2,000, respectively (Supplementary Table S2). And the setting of hyperparameters is almost the same way as the Xu’s paper (Xu, 2010). The MSE is used to evaluate the performance of ScoreEB under various hyperparameter values. And results show that the MSE is minimum when (τ,ω) is set to (0,0) at sample size 200, 500 and 2,000. It means that (τ,ω)=(0,0) (the Jeffrey’s prior) is the best choice at these three sample sizes. Only when sample size is 1,000, (τ,ω)=(0.5,0) is the best choice with minimum MSE 0.00167. At this time, the MSE of (τ,ω)=(0,0) is 0.00173, which is slightly larger than that of (τ,ω)=(0.5,0) (Supplementary Table S2). It should be noted that Empirical Bayes is a component of ScoreEB, and the choice of hyperparameters is different from the direct use of Empirical Bayes. Our results demonstrate that (τ,ω)=(0,0) (the Jeffrey’s prior) is robust and almost the best at different sample size.

Complex genetic architecture plays a key role in influencing the statistical power, which often leads single-locus methods to perform poorly. However, multi-locus methods can identify and account for complex genetic architectures, such as, allelic heterogeneity, and rare variant architecture (Korte and Farlow, 2013). Interestingly, genetic heterogeneity can lead to a non-causative marker being a better descriptor of the phenotype than a causative one (Platt et al., 2010). One available approach is fitting multiple SNPs in a genomic region into multi-locus mixed model, in this case, it may consider allelic heterogeneity. Another common issue is rare variant architecture, which may not always be resolved by increasing sample size. One solution is to collapse several SNPs in a region into a single indicator variable and use this as a composite genotype (Feng and Zhu, 2012). Therefore, solving complex genetic structure problems is another important motivation to develop ScoreEB.

Although we found that ScoreEB is an efficient and powerful multi-locus method, our approach is not free of limitations. ScoreEB is currently only suitable for analyzing quantitative traits, and is not available for analysis of binary traits. Binary traits are common, for example, stress tolerance in plants and case-control in human beings, and are mostly based on logistic or generalized linear models. ScoreEB detected a small number of genes also identified by the other methods (Supplementary Table S3 and Figure 3). Although the multi-locus methods ScoreEB, mrMLM, FASTmrEMMA, ISIS EM-BLASSO and HRePML perform relatively well in simulation studies, their consistency in real data analysis is not satisfactory. It is accepted that complementarity exists between different multi-locus GWAS methods (Ren et al., 2018). At present, ScoreEB has a very high computational efficiency, when the number of individuals *N* is not very large (*n* < 3,000), such as, most plant researches. For researches with millions of individuals, we recommend BOLT-LMM (Loh et al., 2015) or fastGWA (Jiang et al., 2019). In response to these limitations, we will continue to improve ScoreEB in future work. These improvements will include: 1) Extend the approach to analyze binary trait *via* a link function. 2) Further explore the issue of fewer identical genes being identified compared to different methods.

## Conclusion

In this paper, we demonstrated that ScoreEB is a fast and powerful GWAS method for quantitative trait analysis. In addition, ScoreEB has the ability to accurately estimate the QTN effect and effectively control the false positive rate. Using ScoreEB analysis can contribute to increasing our knowledge of the underlying mechanisms of complex traits and to predicting more candidate genes for molecular assisted breeding.

## Data Availability

Publicly available datasets were analyzed in this study. The *Arabidopsis* dataset can be found in Tair http://www.arabidopsis.org/, the rice dataset can be found in Rice Diversity http://www.ricediversity.org/, the maize dataset can be found in Panzea https://www.panzea.org/, the cattle dataset can be found in https://www.g3journal.org/content/5/4/615.supplemental, the pig dataset can be found in https://figshare.com/articles/pig-growth-data_zip/7533020. The R code implementation of ScoreEB and simulation datasets are available on https://github.com/wenlongren/ScoreEB. The ScoreEB package is also maintained on https://cran.r-project.org/web/packages/ScoreEB/index.html.
