# Supplementary material for: An Efficient Score Test Integrated with Empirical Bayes for Genome-Wide Association Studies
Source: Front Genet. 2021 Oct 1;12:742752. doi: 10.3389/fgene.2021.742752 (PMC8517403; doi:10.3389/fgene.2021.742752)
Supplement: Supplementary file 1 [file Presentation1.pdf]

Appendix

A1. Derivation of the Score Test Statistic

Based on the following log likelihood function,

$$L(\theta) = -\frac{n}{2} log\left(2\pi\right) - \frac{1}{2} log\left|\Sigma\right| - \frac{1}{2}\left\{\left(\boldsymbol{y} - \boldsymbol{Xb}\right)^{T} \Sigma^{-1} \left(\boldsymbol{y} - \boldsymbol{Xb}\right)\right\}$$

the score test statistic with σ_g^2 scalar is written as

$$\begin{aligned}
T_{score} &= \frac{\partial L(\sigma_g^2, \hat{\boldsymbol{b}}, \widehat{\sigma_k^2}, \widehat{\sigma_e^2})}{\partial \sigma_g^2} \\
&= \frac{\partial\left[-\frac{n}{2} log\left(2\pi\right)\right]}{\partial \sigma_g^2} - \frac{1}{2}\frac{\partial log\left|\Sigma\right|}{\partial \sigma_g^2} - \frac{1}{2}\frac{\partial\left\{\left(\boldsymbol{y} - \boldsymbol{X}\hat{\boldsymbol{b}}\right)^{T} \Sigma^{-1} \left(\boldsymbol{y} - \boldsymbol{X}\hat{\boldsymbol{b}}\right)\right\}}{\partial \sigma_g^2} \\
&= 0 - \frac{1}{2}\frac{Tr(\Sigma^{-1}\partial\Sigma)}{\partial \sigma_g^2} - \frac{1}{2}\frac{\left(\boldsymbol{y} - \boldsymbol{X}\hat{\boldsymbol{b}}\right)^{T} \partial\Sigma^{-1} \left(\boldsymbol{y} - \boldsymbol{X}\hat{\boldsymbol{b}}\right)}{\partial \sigma_g^2} \\
&= -\frac{1}{2}\frac{\partial Tr(\Sigma^{-1}\Sigma)}{\partial \sigma_g^2} - \frac{1}{2}\left(\boldsymbol{y} - \boldsymbol{X}\hat{\boldsymbol{b}}\right)^{T} \frac{\partial\Sigma^{-1}}{\partial \sigma_g^2} \left(\boldsymbol{y} - \boldsymbol{X}\hat{\boldsymbol{b}}\right) \\
&= -\frac{1}{2}\frac{\partial Tr(\boldsymbol{I})}{\partial \sigma_g^2} - \frac{1}{2}\left(\boldsymbol{y} - \boldsymbol{X}\hat{\boldsymbol{b}}\right)^{T} \frac{-\Sigma^{-1}(\partial\Sigma)\Sigma^{-1}}{\partial \sigma_g^2} \left(\boldsymbol{y} - \boldsymbol{X}\hat{\boldsymbol{b}}\right) \\
&= 0 + \frac{1}{2}\left(\boldsymbol{y} - \boldsymbol{X}\hat{\boldsymbol{b}}\right)^{T} \Sigma^{-1} \frac{\partial\Sigma}{\partial \sigma_g^2} \Sigma^{-1} \left(\boldsymbol{y} - \boldsymbol{X}\hat{\boldsymbol{b}}\right) \\
&= \frac{1}{2}\left(\boldsymbol{y} - \boldsymbol{X}\hat{\boldsymbol{b}}\right)^{T} \Sigma^{-1} \frac{\partial\left(\sigma_g^2 \boldsymbol{xx}^T + \widehat{\sigma_k^2}\boldsymbol{K} + \widehat{\sigma_e^2}\boldsymbol{I}_n\right)}{\partial \sigma_g^2} \Sigma^{-1} \left(\boldsymbol{y} - \boldsymbol{X}\hat{\boldsymbol{b}}\right) \\
&= \frac{1}{2}\left(\boldsymbol{y} - \boldsymbol{X}\hat{\boldsymbol{b}}\right)^{T} \Sigma^{-1}\boldsymbol{xx}^T\Sigma^{-1} \left(\boldsymbol{y} - \boldsymbol{X}\hat{\boldsymbol{b}}\right)
\end{aligned}$$

Under the null model via maximum likelihood estimates (MLE), formula $\hat{\boldsymbol{b}} = \left(\boldsymbol{X}^T\boldsymbol{M}_0^{-1}\boldsymbol{X}\right)^{-1}\boldsymbol{X}^T\boldsymbol{M}_0^{-1}\boldsymbol{y}$ and $\boldsymbol{M}_0 = \widehat{\sigma_k^2}\boldsymbol{K} + \widehat{\sigma_e^2}\boldsymbol{I}_n$ can be easily obtained. Then, the score test statistic can be further written as

$$\begin{aligned}
T_{score} &= \frac{1}{2}\left(\boldsymbol{y} - \boldsymbol{X}\hat{\boldsymbol{b}}\right)^{T} \boldsymbol{M}_0^{-1}\boldsymbol{xx}^T\boldsymbol{M}_0^{-1} \left(\boldsymbol{y} - \boldsymbol{X}\hat{\boldsymbol{b}}\right) \\
&= \frac{1}{2}\left[\left(\boldsymbol{y} - \boldsymbol{X}\hat{\boldsymbol{b}}\right)^{T} \boldsymbol{M}_0^{-1}\boldsymbol{x}\right]\left[\left(\boldsymbol{y} - \boldsymbol{X}\hat{\boldsymbol{b}}\right)^{T} \boldsymbol{M}_0^{-1}\boldsymbol{x}\right]^T
\end{aligned}$$

$$\because \left(\boldsymbol{y}-\boldsymbol{X}\hat{\boldsymbol{b}}\right)^T \boldsymbol{M}_0^{-1}\boldsymbol{x} = \boldsymbol{y}^T\boldsymbol{M}_0^{-1}\boldsymbol{x} - \hat{\boldsymbol{b}}^T\boldsymbol{X}^T\boldsymbol{M}_0^{-1}\boldsymbol{x}$$

$$= \boldsymbol{y}^T\boldsymbol{M}_0^{-1}\boldsymbol{x} - \left[\left(\boldsymbol{X}^T\boldsymbol{M}_0^{-1}\boldsymbol{X}\right)^{-1}\boldsymbol{X}^T\boldsymbol{M}_0^{-1}\boldsymbol{y}\right]^T\boldsymbol{X}^T\boldsymbol{M}_0^{-1}\boldsymbol{x}$$

$$= \boldsymbol{y}^T\boldsymbol{M}_0^{-1}\boldsymbol{x} - \left[\boldsymbol{y}^T\boldsymbol{M}_0^{-1}\boldsymbol{X}\left(\boldsymbol{X}^T\boldsymbol{M}_0^{-1}\boldsymbol{X}\right)^{-1}\right]\boldsymbol{X}^T\boldsymbol{M}_0^{-1}\boldsymbol{x}$$

$$= \boldsymbol{y}^T\left(\boldsymbol{M}_0^{-1} - \boldsymbol{M}_0^{-1}\boldsymbol{X}\left(\boldsymbol{X}^T\boldsymbol{M}_0^{-1}\boldsymbol{X}\right)^{-1}\boldsymbol{X}^T\boldsymbol{M}_0^{-1}\right)\boldsymbol{x}$$

$$= \boldsymbol{y}^T\boldsymbol{P}\boldsymbol{x}$$

Here, let $\boldsymbol{P} = \boldsymbol{M}_0^{-1} - \boldsymbol{M}_0^{-1}\boldsymbol{X}\left(\boldsymbol{X}^T\boldsymbol{M}_0^{-1}\boldsymbol{X}\right)^{-1}\boldsymbol{X}^T\boldsymbol{M}_0^{-1}$

$$\therefore \quad T_{score} = \frac{1}{2}\boldsymbol{y}^T\boldsymbol{P}\boldsymbol{x}[\boldsymbol{y}^T\boldsymbol{P}\boldsymbol{x}]^T$$

$$= \frac{1}{2}\left\|\boldsymbol{x}^T\boldsymbol{P}\boldsymbol{y}\right\|^2$$

A2. Derivation of the QTN effect in the first simulation study

$$\because \quad h_i^2 = \frac{\sigma_i^2}{\sum_{i=1}^{6}\sigma_i^2 + \sigma_e^2 + \sigma_k^2} = \frac{\sigma_i^2}{\sigma_g^2 + \sigma_e^2 + \sigma_k^2} \qquad \text{(i)}$$

$$h_t^2 = \frac{\sigma_g^2}{\sigma_g^2 + \sigma_e^2 + \sigma_k^2} \qquad \text{(ii)}$$

$$\sigma_i^2 = \left[1-\left(\eta_i-\left(1-\eta_i\right)\right)^2\right]\beta_i^2 \qquad \text{(iii)}$$

$$\therefore \quad \beta_i = \sqrt{\frac{\left[\frac{h_t^2\left(\sigma_e^2+\sigma_k^2\right)}{1-h_t^2}+\sigma_e^2+\sigma_k^2\right]h_i^2}{4\eta_i\left(1-\eta_i\right)}} \qquad \text{(iv)}$$

Here, $\sigma_i^2, h_i^2, \beta_i$ and $\eta_i\left(i=1,\cdots,6\right)$ are the genetic variance, heritability, effect and the minor allele frequency of each QTN, respectively. σ_g^2 and h_t^2 are the total genetic variance and total heritability of all six QTNs, σ_e^2 is the residual variance, and σ_k^2 is the polygenic variance.
