# Supplementary material for: An Efficient Score Test Integrated with Empirical Bayes for Genome-Wide Association Studies
Source: Front Genet. 2021 Oct 1;12:742752. doi: 10.3389/fgene.2021.742752 (PMC8517403; doi:10.3389/fgene.2021.742752)
Supplement: Supplementary file 2 [file DataSheet1.docx]

**Table S1.** Comparison of mean squared errors (MSE) for each QTN among ScoreEB, mrMLM, FASTmrEMMA, ISIS EM-BLASSO, HRePML and GEMMA methods in the second simulation study^*^.

| **QTN** | **Chr.** | **Pos. (bp)** | **R^2^** | **Effect** |  | **Mean Squared Errors (MSE)** | | | | | | |
| --- | --- | --- | --- | --- | --- | --- | --- | --- | --- | --- | --- | --- |
|  |  |  |  |  |  | **ScoreEB** | **mrMLM** | **FASTmrEMMA** | **ISIS EM-BLASSO** | **HRePML** | **GEMMA** |  |
| 1 | 1 | 11,298,364 | 0.10 | 1.4762 |  | 0.1176 | 0.0964 | 0.3018 | 0.1449 | 0.2765 | 0.3048 |  |
| 2 | 2 | 5,134,228 | 0.15 | 1.8080 |  | 0.1846 | 0.1728 | 0.3803 | 0.1874 | 0.4973 | 0.2152 |  |
| 3 | 2 | 5,066,968 | 0.05 | 1.0438 |  | 0.0775 | 0.1362 | 0.5058 | 0.0725 | 0.0515 | 9.1484 |  |
| 4 | 2 | 5,464,675 | 0.05 | 1.0438 |  | 0.0806 | 0.1314 | 0.5840 | 0.0755 | 0.0404 | 9.7825 |  |
| 5 | 2 | 6,137,189 | 0.05 | 1.0438 |  | 0.0532 | 0.0672 | 0.2621 | 0.0778 | 0.0702 | 0.7999 |  |
| 6 | 1 | 11,655,607 | 0.05 | 1.0438 |  | 0.0597 | 0.0784 | 0.2887 | 0.0802 | 0.0684 | 9.3529 |  |
| Average MSE | | | | |  | 0.0955 | 0.1137 | 0.3871 | 0.1064 | 0.1674 | 4.9340 |  |

^*^In the second simulation study, the dataset consists of 199 individuals and 216,130 single nucleotide polymorphism (SNP) markers with 1,000 replicates. Six true QTNs are set in each replicate.

**Table** **S2**. The mean squared error (MSE) of alternative hyperparameters choice for the simulated data set at different sample size.

| **Hyperparameter**  **()** | **** | | | |  | **** | | | |
| --- | --- | --- | --- | --- | --- | --- | --- | --- | --- |
|  | **200** | **500** | **1,000** | **2,000** |  | **200** | **500** | **1,000** | **2,000** |
| -5 | 0.014237504 | 0.003517128 | **0.001673196** | 0.000459997 |  | 6.108974161 | 1.443075222 | 0.007679142 | 0.005282408 |
| 0 | **0.012352543** | **0.003350595** | 0.001733737 | **0.000427501** |  | **0.017302144** | **0.00389809** | **0.001940865** | **0.000553853** |
| 0.5 | 0.01300866 | 0.003428952 | 0.001727686 | 0.000437167 |  | 0.044598536 | 0.02371691 | 0.007128852 | 0.004027097 |
| 1 | 0.013998535 | 0.003456341 | 0.001737539 | 0.000444001 |  | 0.111100974 | 0.039473796 | 0.007306538 | 0.004106942 |
| 2 | 0.014706798 | 0.00357478 | 0.001770434 | 0.000462157 |  | 0.175886894 | 0.113042117 | 0.007402956 | 0.004145699 |
| 3 | 0.015323518 | 0.003625946 | 0.001866486 | 0.000485777 |  | 0.182823616 | 0.286440162 | 0.007435108 | 0.004156317 |
| 4 | 0.016369091 | 0.003733915 | 0.00189237 | 0.000515726 |  | 0.190231081 | 0.297331438 | 0.007450177 | 0.004160574 |
| 5 | 0.017302144 | 0.00389809 | 0.001940865 | 0.000553853 |  | 0.199062386 | 0.305482125 | 0.007458572 | 0.004162309 |
| 6 | 0.018139073 | 0.004128821 | 0.002003414 | 0.000592473 |  | 0.210321785 | 0.316789585 | 0.007463749 | 0.004163071 |
| 7 | 0.019640895 | 0.004372963 | 0.002098131 | 0.000633566 |  | 0.225488832 | 0.334779244 | 0.007467165 | 0.004162951 |
| 8 | 0.020525226 | 0.004636719 | 0.002201911 | 0.000690558 |  | 0.248125851 | 0.374557054 | 0.00746953 | 0.004162626 |
| 9 | 0.016945339 | 0.005013743 | 0.002313509 | 0.000752228 |  | 0.291532456 | 0.475419337 | 0.007471229 | 0.004162205 |
| 10 | 0.018562845 | 0.005389899 | 0.00243561 | 0.000822719 |  | 0.444156541 | 0.480540264 | 0.007472484 | 0.004161743 |
| 15 | 0.024060939 | 0.007647697 | 0.003613195 | 0.001430506 |  | 0.455647734 | 0.494413455 | 0.007475548 | 0.004159494 |
| 50 | 0.236519394 | 0.172651367 | 0.536738982 | 0.429617331 |  | 0.455464975 | 0.502594759 | 0.007477154 | 0.004152823 |

**Table S3.** Previously reported genes that were identified in *Arabidopsis*, rice, maize, cattle and pig at least by four methods simultaneously with ScoreEB, mrMLM, FASTmrEMMA, ISIS EM-BLASSO, HRePML and GEMMA^*^.

| **Species** | **Chr.** | **Position** | **Lod or P value** | **Nearby Genes or QTLs** | **Methods** |
| --- | --- | --- | --- | --- | --- |
| *Arabidopsis* | 4 | 268,990 | 8.26, 15.90, 15.56, 6.59 | *KEA2* | 1, 3, 4, 5 |
|  | 4 | 268,990/ 276,143/ 268,990/ 268,990/ 268,990/ 269,260 | 8.26, 20.08, 15.90, 15.56, 6.59, 1.43×10^-17^ | *FLA* | 1, 2, 3, 4, 5, 6 |
|  | 4 | 276,143/ 276,143/ 276,143/ 275,349 | 20.08, 9.29, 3.59, 6.80×10^-18^ | *ATRH8* | 2, 4, 5, 6 |
| Maize | 1 | 244,827,585 | 4.52, 8.41, 13.43, 6.43, 7.29 | *GRMZM2G164761* | 1, 2, 3, 4, 5 |
|  | 5 | 5,791,489 | 5.04, 7.17, 3.62, 4.27 | *GRMZM2G104789* | 2, 3, 4, 5 |
|  | 5 | 215,479,655 | 6.25, 10.76, 8.49, 3.64 | *GRMZM5G807336* | 1, 2, 3, 4 |
|  | 6 | 129,495,428 | 3.10, 5.17, 7.84, 4.54 | *GRMZM2G174315* | 1, 2, 3, 5 |
|  | 6 | 148,245,602 | 4.08, 10.89, 15.41, 7.75 | *GRMZM5G835629* | 1, 2, 3, 5 |
|  | 6 | 158,480,724 | 3.25, 4.48, 4.47, 3.02 | *GRMZM2G425230* | 1, 2, 3, 5 |
|  | 7 | 2,176,113/ 2,176,113/2,176,113/ 2,160,710 | 3.56, 7.24, 4.94, 3.08 | *GRMZM2G072582* | 2, 3, 4, 5 |
|  | 7 | 156,014,666 | 7.54, 7.93, 7.69, 3.17 | *GRMZM2G032253* | 1, 2, 3, 4 |
|  | 7 | 173,186,109 | 6.05, 5.35, 7.28, 4.55 | *GRMZM2G136872* | 1, 2, 3, 4 |
|  | 8 | 114,400,659 | 11.69, 15.92, 4.95, 14.91 | *GRMZM2G053882* | 2, 3, 4, 5 |
|  | 8 | 123,509,373/ 123,512,730/ 123,512,155/ 123,509,373/ 123,512,155 | 5.98, 3.64, 4.68, 7.45, 12.89 | *GRMZM2G083016* | 1, 2, 3, 4, 5 |
|  | 9 | 8,237,062 | 3.37, 13.90, 11.78, 4.48, 4.11 | *GRMZM2G469489* | 1, 2, 3, 4, 5 |
|  | 9 | 153,214,218 | 5.94, 5.47, 10.01, 4.22 | *GRMZM2G086934* | 1, 2, 3, 5 |
|  | 10 | 3,185,640 | 4.97, 5.65, 12.35, 4.98, 5.89 | *GRMZM2G069382* | 1, 2, 3, 4, 5 |
|  | 10 | 6,375,466 | 9.26, 11.92, 8.95, 10.66 | *GRMZM2G052499* | 1, 2, 3, 5 |
|  | 10 | 145,014,181 | 3.45, 6.01, 9.38, 3.10 | *GRMZM2G029242* | 1, 2, 3, 4 |
|  | 10 | 147,890,728 | 3.46, 5.20, 4.62, 4.12 | *GRMZM2G153096* | 2, 3, 4, 5 |
| Cattle | 5 | 70,895,855/ 70,836,824/ 70,954,886/ 70,895,855/ 70,895,855 | 6.13, 10.76, 6.27, 6.45, 23.06 | *BTBD11* | 1, 2, 3, 4, 5 |
|  | 5 | 82,997,146 | 6.13, 5.42, 9.07, 5.65 | *STK38L* | 1, 2, 4, 5 |
|  | 14 | 1,610,986 | 241.03, 20.09, 19.97, 135.96, 7.25×10^-92^ | *VPS28* | 1, 2, 3, 4, 6 |
|  | 16 | 1,889,480 | 6.03, 6.40, 5.86, 6.17 | *PIK3C2B* | 2, 3, 4, 5 |
|  | 17 | 74,864,792 | 6.41, 3.25, 6.35, 5.28 | *ARVCF* | 1, 2, 4, 5 |
|  | 18 | 39,611,807 | 4.18, 4.52, 10.03, 12.77 | *CHST4* | 1, 2, 3, 5 |
|  | 20 | 14,703,246 | 11.23, 5.22, 4.32, 9.04 | *SHISAL2B* | 2, 3, 4, 5 |
|  | 27 | 34,249,415 | 7.67, 7.37, 11.21, 4.99 | *ADAM2* | 1, 2, 4, 5 |
| Pig | 1 | 57,487,161/ 57,399,350/ 57,487,161/ 57,399,350/ 57,487,161 | 8.44, 5.16, 7.10, 11.65, 5.48 | *ANKRD6* | 1, 2, 3, 4, 5 |
|  | 1 | 161,987,727 | 13.41, 7.79, 14.25, 20.72 | *MALT1* | 1, 3, 4, 5 |
|  | 5 | 80,393,072 | 3.72, 10.85, 4.83, 6.45 | *TDG* | 1, 3, 4, 5 |
|  | 6 | 93,010,249/ 92,923,935/ 92,923,935/ 92,923,935/ 92,923,935 | 3.87, 6.69, 7.60, 5.28, 12.88 | *GRIK3* | 1, 2, 3, 4, 5 |
|  | 8 | 3,371,469 | 6.06, 3.59, 4.60, 4.43 | *SORCS2* | 1, 2, 3, 5 |
|  | 13 | 24,433,904 | 5.84, 6.01, 4.19, 4.75, 4.38 | *MYRIP* | 1, 2, 3, 4, 5 |

^*^ Use 1, 2, 3, 4, 5 and 6 to represent ScoreEB, mrMLM, FASTmrEMMA, ISIS EM-BLASSO, HRePML and GEMMA methods, respectively. There are no genes identified by at least four methods simultaneously in rice.
